# Identification and characterization of 3-ketosphinganine reductase activity encoded at the *BT_0972* locus in *Bacteroides thetaiotaomicron*

**DOI:** 10.1016/j.jlr.2022.100236

**Published:** 2022-06-03

**Authors:** Min-Ting Lee, Henry H. Le, Kevin R. Besler, Elizabeth L. Johnson

**Affiliations:** Division of Nutritional Sciences, Cornell University, Ithaca, New York, USA

**Keywords:** bacterial sphingolipids, sphinganine, 3-ketosphinganine reductase, *Bacteroides thetaiotaomicron*, metabolomics, host-microbe interactions, BT_0972, gut microbiome, enzymatic assay, sphingolipid synthesis, 3-KDS, 3-ketosphinganine, 3-KDS^alk^, 3-ketosphinganine alkyne, 3-KDSR, 3-ketosphinganine reductase, Bt, *B. thetaiotaomicron*, LB, Luria broth, LC-MS, liquid chromatography-mass spectrometry, PA^alk^, palmitic acid alkyne, SA, sphinganine, SPT, serine palmitoyl transferase, SA^alk^, sphinganine alkyne

## Abstract

Bacterial sphingolipid synthesis is important for the fitness of gut commensal bacteria with an implied potential for regulating mammalian host physiology. Multiple steps in bacterial sphingolipid synthesis pathways have been characterized previously, with the first step of de novo sphingolipid synthesis being well conserved between bacteria and eukaryotes. In mammals, the subsequent step of de novo sphingolipid synthesis is catalyzed by 3-ketosphinganine reductase, but the protein responsible for this activity in bacteria has remained elusive. In this study, we analyzed the 3-ketosphinganine reductase activity of several candidate proteins in *Bacteroides thetaiotaomicron* chosen based on sequence similarity to the yeast 3-ketosphinganine reductase gene. We further developed a metabolomics-based 3-ketosphinganine reductase activity assay, which revealed that a gene at the locus *BT_0972* encodes a protein capable of converting 3-ketosphinganine to sphinganine. Taken together, these results provide greater insight into pathways for bacterial sphingolipid synthesis that can aid in future efforts to understand how microbial sphingolipid synthesis modulates host-microbe interactions.

Sphingolipids are important bioactive signaling molecules associated with various cellular processes, including cell proliferation, differentiation, and apoptosis (1). Although sphingolipid production is ubiquitous in eukaryotes, only a subset of bacteria is known to make sphingolipids (2, 3). This subset comprises members of the Bacteroidetes phylum which is inclusive of the genera *Bacteroides*, *Prevotella*, *Porphyromonas*, and *Sphingobacterium* (3, 4, 5, 6). For the *Bacteroides* genus, a common member of the intestinal microbiota of healthy individuals (7, 8), sphingolipids comprise as much as 70% of the membranous phospholipid content (9, 10). Several studies have identified host-specific bioactivities associated with bacterially derived sphingolipids, including modulation of host immunity (9, 10, 11, 12, 13, 14) and metabolism (15), suggesting that bacterial sphingolipid synthesis is important to host-microbiome interactions. Although sphingolipid biosynthesis is well characterized for eukaryotic systems (1), knowledge of these processes in bacterial systems is sparce. (14). Thus, identifying and characterizing genes encoding key enzymes in microbial sphingolipid biosynthetic pathways will facilitate the analysis and discovery of sphingolipid-dependent phenomena for bacteria and host-microbiome interactions.

*De novo* biosynthesis of canonical sphingolipids begins with the condensation of an amino acid (typically *L*-serine in eukaryotes) and a fatty acid (typically palmitate in mammals) to generate 3-ketosphinganine (3-KDS, 3-ketodihydrosphingosine) by serine palmitoyl transferase (SPT) (1, 16). 3-ketosphinganine reductase (3-KDSR) then converts 3-KDS to sphinganine (SA), which can be N-acylated to produce dihydroceramide, a key intermediate to more complex sphingolipids (9, 17). Although a diverse array of sphingolipids have been detected in *Bacteroides* (10), the identification of genes responsible for these transformations is limited to enzymes with the activity of SPT (18, 19, 20), SA kinase (9), ceramide synthase (21), and branched chain amino acid aminotransferase (14). Manipulation of SPT (10, 13) and branched chain amino acid aminotransferase (14) activities in mouse microbiomes have been shown to modulate host immunity (10, 13, 14) and sphingolipid metabolism (15). These outcomes indicate that the ability to control bacterial sphingolipid metabolism may serve as a therapeutic tool.

Although characterized by Dunn et al. in *Saccharomyces cerevisiae* (*TSC10*) in 1998 (22) and the human homolog (*FVT-1*) in 2004 (23), the bacterial gene for 3-KDSR remains unidentified. Recently, it was suggested that bacteria forego KDSR activity with the discovery of dihydroceramides with unreduced SA backbones in *Caulobacter crescentus* (21), an environmental bacterium able to produce sphingolipids (24). However, we present evidence for diverged sphingolipid synthesis strategies in bacteria with the characterization of 3-KDSR in *Bacteroides thetaiotaomicron.* This is the first definitive characterization of 3-KDSR activity in a bacterial sphingolipid synthesis pathway. In this work, via comparative genomics, biochemical- and liquid chromatography-mass spectrometry (LC-MS)–based methods, we identify the operative 3-KDSR in the common human gut commensal *B. thetaiotaomicron.*

## Materials and methods

### Identification of KDSR candidate genes in *B. thetaiotaomicron*

To identify the *kdsr* locus within the genome of *B. thetaiotaomicron*, amino acid sequences of KDSR in *S. cerevisiae* (*TSC10*, codes for a KDSR) and the human homolog *FVT1* were used to BLASTp search against *B. thetaiotaomicron* using the default search tool in the Kyoto Encyclopedia of Genes and Genomes (KEGG, https://www.kegg.jp/). The top seven hits with putative oxidoreductase annotation were selected as KDSR candidate genes in *B. thetaiotaomicron*.

### Bacterial strains and culturing

*B. thetaiotaomicron* strain VPI 5482 was cultured in either brain heart infusion medium or minimal medium, consisting of 13.6 g KH_2_PO_4_, 0.875 g NaCl, 1.125 g (NH_4_)_2_SO_4_, 5 g glucose (pH to 7.2 with concentrated NaOH), 1 ml hemin solution (500 mg dissolved in 10 ml of 1M NaOH then diluted to a final volume of 500 ml with water), 1 ml MgCl_2_ (0.1 M in water), 1 ml FeSO_4・_7H_2_O (1 mg per 10 ml of water), 1 ml vitamin K3 (1 mg/ml in absolute ethanol), 1 ml CaCl_2_ (0.8% w/v), 250 μl vitamin B12 solution (0.02 mg/ml), and 5 g L-cysteine hydrochloride anhydrous. The medium was prepared freshly and prereduced in an anaerobic chamber (gas mix: 70% N_2_, 25% CO_2_, and 5% H_2_).

### Cloning and expression of KDSR candidate genes in *B. thetaiotaomicron*

Candidate 3-KDSR genes (supplemental Table S1) were amplified from the genome of *B. thetaiotaomicron* (*Bt*) and cloned into XhoI and NcoI-linearized pET-28a (EMD Millipore, Burlington, MA). *TSC10* was amplified from the genome of *S. cerevisiae* (BY4741) and cloned into XhoI and NcoI-linearized pET-28a (EMD Millipore. The assembled constructs were transformed into *Escherichia coli* NEB 5-alpha (New England Biolabs) chemically competent cells and plated onto Luria broth (LB) agar plates containing kanamycin (50 μg/ml). *Bt*SPT was amplified and cloned into XhoI and NdeI linearized pET-21a (EMD Millipore). The assembled construct was transformed into (*E. coli)* NEB 5-alpha chemically competent cells and plated onto LB agar plates containing carbenicillin (100 μg/ml). All colonies were PCR-screened for successful assembly and Sanger sequenced. The candidate 3-KDSR plasmid (or TSC10 plasmid) and the *Bt*SPT plasmid were cotransformed into BL21 *E. coli* and plated onto agar plates containing kanamycin (50 μg/ml) and carbenicillin (100 μg/ml). BL21 *E. coli* double transformants were grown overnight in LB with kanamycin (50 μg/ml) and carbenicillin (100 μg/ml). Overnight cultures were diluted (1:100) into fresh LB-containing antibiotics and 25 μM palmitic acid alkyne (PA^alk^) and 100 μM serine. IPTG was added when the A_600_ reached 0.4 and incubated at 37°C at 250 RPM. The reactions were monitored over a time course by sampling 1 ml out of the total reaction mix (5 ml) at each of the following time points post adding IPTG: 0 min, 30 min, 4 h, and 24 h. The samples (duplicates for each candidate gene) were snap frozen using liquid nitrogen to stop the reaction at the time of collection and stored at −80°C. After completing the collection, samples were lyophilized for two days, and metabolome extractions were prepared as described below for further identification of KDSR function.

### Expression and enrichment of proteins in this study

The *Bt*SPT was prepared as explained by Wadsworth *et al.* (25). *BT_0972* was PCR-amplified with primers pET28_BT0972_fwd and pET28_BT0972_rev listed in supplemental Table S1 from the *B. thetaiotaomicron* genome, and the products were cloned into XhoI and NcoI linearized pET21a. The resulting construct was transformed into *E. coli* BL21, plated onto LB agar-kanamycin (50 μg/ml) plates, and screened for successful integration of the plasmid. Stater cultures of transformants were grown overnight, inoculated into 1 L of LB media containing kanamycin (50 μg/ml), and incubated at 37°C on a rotary shaker at 250 RPM. Cultures were induced at A_600_ = 0.6 with 1 mM IPTG and incubated on a shaker for 5 h at 37°C at 250 RPM. Cells were then harvested via centrifugation and frozen overnight. Thawed cells were resuspended in lysis buffer (25 mM Tris-HCl, pH 7.5, 300 mM NaCl, 10 mM imidazole, 10 mM 2-mercaptoethanol, 10% glycerol, 1% Triton X-100, 1 mg/ml lysozyme, and 1x ProBlock Gold Bacterial 2D Protease Inhibitor Cocktail (GoldBio)). Cells were sonicated in 30 s pulses for 2 min total and centrifuged for 20 min at 18,213 *g*. The cleared lysate was moved to a new tube and incubated with 2 ml of Ni-NTA agarose beads for 30 min on a rocking platform. The lysate and Ni-NTA agarose were loaded onto a 1.5 cm column, washed with lysis buffer containing 20 mM and 40 mM imidazole, and eluted with a buffer containing 350 mM imidazole. After the determining protein concentration (2.5 mg/ml), the eluate was flash frozen using liquid nitrogen and stored at −80°C until usage. SDS-PAGE and Coomassie staining were performed to confirm the size of the purified protein of *BT_0972* (BT_0972p). A control purification (mock purification) was performed in parallel with identical conditions using *E. coli* BL21 pET21a_empty.

### Enzymatic synthesis of 3-KDS

3-KDS was chemically synthesized with assay conditions comprising of acyl-CoA synthesis (26, 27) followed by condensation with *L*-serine: 50 mM potassium phosphate pH 7.5, 10 mM adenosine triphosphate, 2 mM coenzyme A, 5 mM magnesium chloride, 0.05% (v/v) Tween 20, 250 μM PA^alk^ (Click Chemistry Tools, Scottsdale, AZ) or palmitic acid (MP Bio), and 0.025 U of acyl-coenzyme A synthetase from *Pseudomonas* sp.(Sigma Aldrich) to a final volume of 1 ml and incubated at 37°C for 2 h. A final concentration of 2 mM of *L*-serine (Sigma Aldrich) was added along with 1 μl of *Bt*SPT-His6 solution and incubated for three additional hours at 35°C. Ten microliters of each 1 ml reaction was aliquoted for LC-MS analysis (described below) for confirming the successful formation of 3-KDS.

### Verification of 3-KDSR activity by purified bacterial protein conversion of 3-KDS to SA

Two hundred microliters of the above prepared 3-KDS enzymatic reactions were incubated with 10 μl BT_0972p (2.5 mg/ml) or equal volumes of empty vector protein at 37°C for 24 h under the following reaction conditions: 250 μM NADPH or NADH, 2.5 mM glucose 6-phosphate, and 18 U glucose-6-phosphate dehydrogenase. The resulting solution was briefly spun down and immediately frozen with liquid nitrogen, lyophilized to dryness, and underwent metabolite extraction and LC-MS analysis as described below.

### Metabolome preparation for LC-MS analysis

To extract metabolites from samples, 1 ml of methanol was added to the dried materials for all the samples of metabolomic analysis. Additionally, 1.5 μl of 20 μM sphinganine-d7 (Avanti Lipids, Alabaster, AL) was added to samples for targeted metabolomics as an internal standard. Samples were sonicated for 3 min with on/off cycles of three seconds on and two seconds off at 100% power on a Qsonica’s Ultrasonic Processor with the Cup Horn water bath attachment maintained at 20°C. The samples were then placed on an end-over-end rotator for overnight extraction. Samples were then centrifuged at 18,000 *g* at 4°C for 30 min. The clarified supernatant was collected and transferred to a fresh 1.7 ml centrifuge tube, dried using a SpeedVac vacuum concentrator (Thermo Fisher Scientific, Waltham, MA), and then reconstituted in 200 μl of methanol. Samples were sonicated again and then centrifuged at 18,000 *g* at 4°C for 30 min. One hundred and fifty microliters of clarified concentrated extracted metabolome was transferred to an HPLC vial utilizing an insert (Thermo Fisher Scientific, Waltham, MA) and stored at 4°C until LC-MS analysis.

### LC-MS analysis for targeted metabolomics

Initial studies confirming production of 3-KDS were performed on a Thermo Fisher Scientific Vanquish Horizon UHPLC System coupled with a Thermo Q Exactive HF high resolution mass spectrometer with a HESI ion source. One microliter of extract was injected and separated using a water-acetonitrile gradient on a Kinetex EVO C18 column (150 mm × 2.1 mm, particle size 1.7 μm, part number: 00F-4726-AN) maintained at 40°C. Solvent A: 0.1% formic acid in water; solvent B: 0.1% formic acid in acetonitrile. A/B gradient started at 10% B for 3 min after injection and increased linearly to 100% B at 17 min and held at 100% B for 10 min, using a flow rate 0.5 ml/min. Mass spectrometer parameters: spray voltage 3.5 kV for positive mode and 3.0 kV for negative mode; capillary temperature, 380°C; prober heater temperature, 400°C; sheath, auxiliary, and spare gas: 60, 20, and 1, respectively; S-lens RF level, 50; resolution, 240,000 at m/z 200; AGC target, 3 × 106. Samples were analyzed in full scan positive mode with m/z range 100–1000.

Subsequent LC-MS analysis was performed on a Thermo Fisher Scientific Vanquish Horizon UHPLC System coupled with a Thermo Fisher Scientific TSQ Quantis Triple Quadrupole mass spectrometer equipped with a HESI ion source. Mobile phase A was 78.6% water, 20% acetonitrile, and 0.4% formic acid (v/v). Mobile phase B was 47.8% methanol, 47.8% acetonitrile, 4% chloroform, and 0.4% formic acid. One microliter of extract was injected and separated on a mobile phase gradient with an Agilent Technologies InfinityLab Poroshell 120 EC-C18 column (50 mm × 2.1 mm, particle size 2.7 μm, part number: 699775-902) maintained at 50°C. A/B gradient started at 10% B for 1 min after injection and increased linearly to 100% B at 9 min and held at 100% B for 10 min, using a flow rate 0.6 ml/min. Full Scan Q1 mass spectrometer parameters were as follows: spray voltage, 4.8 kV in positive mode; ion transfer tube temperature, 275°C; vaporizer temperature, 250°C; and sheath, auxiliary, and spare gas: 53.2, 16.3, and 3.3, respectively. The mass spectrometer was calibrated with the Pierce Triple Quadrupole Calibration Solution Extended Mass Range solution. Samples were analyzed in full scan positive mode with m/z range 150–1000, and SA and sphinganine alkyne (SA^alk^) (Click Chemistry Tools, Scottsdale, AZ) were verified with synthetic standards. For determining the relative abundance of 3-KDS alkyne and SA^alk^ from the 24h incubation of the KDSR candidate genes in *B. thetaiotaomicron*, the area under curve of 3-KDS alkyne or SA^alk^ was normalized using the area under curve of the internal standard (SA-d7) for each sample. Values were normalized to the dried weight of the material used for metabolome extraction. Not detected was labeled on the plot if no signal was detected in that sample. Single-reaction monitoring was established by optimizing the method using the SA^alk^ standard and further applied to confirm the detection of SA^alk^ with the following parameters: positive mode; precursor ion: 298.27; product ion: 60.05; collusion energy: 30V; RT window: 0.3 min.

### Comparative sequence analysis of BT_0972 in other known sphingolipid-producing bacteria

The amino acid sequence of BT_0972 was BLASTp searched against a curated sphingolipid-producing bacteria database (1, 2, 3) (also including the 3-KDSR gene in human and mouse as a comparison) using an E-value threshold of 1 × 10^-30^ in the Kyoto Encyclopedia of Genes and Genomes (https://www.kegg.jp/) tool set. All homologs with an E-value of less than 1 × 10^-30^ were selected and the TREE function was applied. Alignment and phylogenetic reconstructions were performed using the function "build" of ETE3 v3.1.1 (28). Alignment was performed with MAFFT v6.861b with the default options (29). The phylogenetic profile was first generated using FastTree v2.1.8 with default parameters (30), and then the output tree file was then used in ggtree R package (31) to construct the tree.

## Results

### Identification of putative 3-KDSR in *B. thetaiotaomicron*

**Fig. 1:**
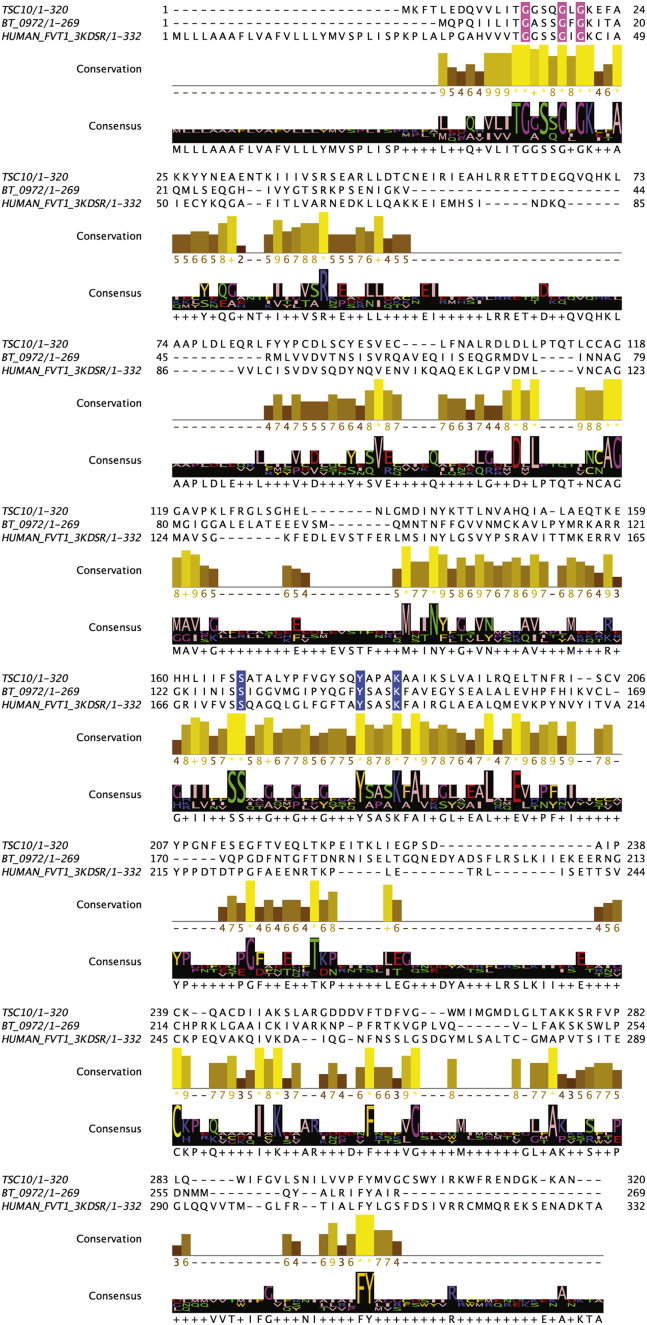
Amino acid sequence comparison of yeast, mammalian, and bacterial KDSR. Amino acid sequence alignment of *Saccharomyces cerevisiae* TSC10, BT_0972, and *Homo sapiens* FVT1 was generated using Jalview software. The putative active site motif, Tyr-*X*-*X*-*X*-Lys (blue) conserved in the short-chain dehydrogenase/reductase (SDR) family and the coenzyme NAD(H) or NADP(H) binding segment Gly-*X*-*X*-*X*-Gly-*X*-Gly (pink) are indicated. KDSR, ketosphinganine reductase.

To identify the putative 3-KDSR in *Bacteroides*, we performed BLASTp of the genome database of *B. thetaiotaomicron* VPI-5482 (32), a genetically manipulable *Bacteroides* strain common to the human gut. We used the amino acid sequence of the *S. cerevisiae* Tsc10p and human FVT-1 (hFVT-1), both of which code for established 3-KDSR enzymes (22, 33), as query sequences. Despite revealing no full homologs, seven partial homologs with pairwise sequence similarity ranging from ∼16% to ∼30% identity (supplemental Fig. S1) were identified. Similar to the yeast (Tsc10p) and human (hFVT-1) 3-KDSR, these candidates belong to the short-chain dehydrogenase/reductase family. Specifically, interrogation of the multiple sequence alignment identified that BT_2380, BT_1433, BT_3771, and BT_0972 included conserved serine, tyrosine, and lysine residues (based on the *S. cerevisiae* numbering in the sequence alignment in supplemental Fig. S2: Ser167, Tyr180, and Lys184) that correspond to the catalytic triad of residues found in SDRs (Figs. 1 and S2, blue) (1, 23, 34). We additionally looked for the conserved consensus of a Gly-*X*-*X*-*X*-Gly-*X*-Gly segment (Figs. 1 and S2, pink) featured in NADPH binding (34) as 3-KDSR activity canonically requires NADPH as a cosubstrate. This sequence was exhibited in three of the four candidates with the exception being BT_1433. Further analysis of the phylogenetic tree suggested that among the four candidates, BT_0972 (Fig. 1) represents the strongest match to the eukaryotic 3-KDSR (supplemental Fig. S3). To confirm 3-KDSR activity, we sought to directly investigate the 3-KDS to SA conversion capacity of each candidate gene.

### BT_0972 is an operative KDSR

**Fig. 2:**
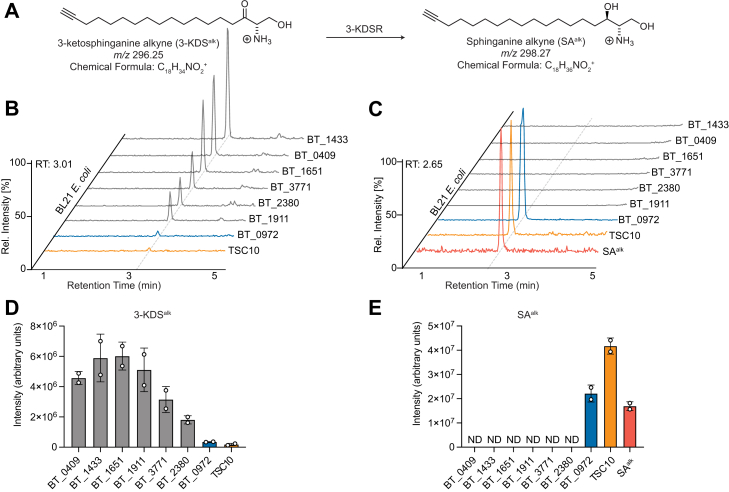
BT_0972 is an operative 3-KDSR in *Bacteroides thetaiotaomicron*. A: Scheme for enzymatic conversion of 3-ketosphinganine alkyne (3-KDS^alk^) to sphinganine alkyne (SA^alk^) catalyzed by 3-ketosphinganine reductase (3-KDSR). Ion chromatograms of (B) 3-KDS^alk^ and (C) SA^alk^ detected in *B. thetaiotaomicron* 3-KDSR candidates that were heterologously expressed in BL21 *Escherichia coli*. Quantification of (D) 3-KDS^alk^ and (E) SA^alk^ detected in *B. thetaiotaomicron* KDSR candidates that were heterologously expressed in BL21 *E. coli*. The *Saccharomyces cerevisiae* 3-KDSR (TSC10) was expressed in BL21 *E. coli* and used as a positive control for 3-KDSR activity. A synthetic SA^alk^ standard was used to detect SA^alk^. (ND, not detected. n = 2 biological replicates per gene candidate and positive controls; representative traces are shown here.) 3-KDS^alk^, 3-ketosphinganine alkyne; 3-KDSR, 3-ketosphinganine reductase; SA^alk^, sphinganine alkyne.

To screen for KDSRs, we developed a system to evaluate KDSR activity. To accomplish this, we required a system which supplied the requisite substrates for KDSR activity, the first being 3-KDS and the second being NADPH. Additionally, we took advantage of the lack of native de novo sphingolipid synthesis in *E. coli*, which allowed us to use a heterologous expression system to assess KDSR activity while avoiding any downstream processing of sphingolipids that would confound observations specific to the conversion of 3-KDS to SA. Specifically, we cloned and expressed the SPT enzyme from *Bt*SPT in BL21 *E. coli* via the pET-28a expression plasmid, a system which we predicted should generate 3-KDS in vivo. Indeed, analysis of metabolomic extracts from *E. coli* harboring *Bt*SPT via liquid chromatography high-resolution mass spectrometry showed the gain of *m/z* 300.28971 (C_18_H_28_NO_2_), consistent with the production of 3-KDS (supplemental Fig. S4). As the pET-28a vector relies on the tunable, albeit leaky lac operon, addition of the inducer IPTG increased production of 3-KDS. The other requisite substrate for 3-KDSR activity is NADPH, a metabolite that is natively supplied by *E. coli* metabolism (35). As a positive control for 3-KDSR activity, we then cotransformed *S. cerevisiae* 3-KDSR (*TSC10*) and confirmed the conversion of 3-KDS to SA (Fig. 2A–C). As SA can also be produced by ceramide hydrolysis (36, 37, 38), a potential contaminant from complex bacterial media, we employed the use of PA^alk^, a labeled analog of PA, and successfully generated SA^alk^ in a *Bt*SPT-TSC10-dependent manner, validating our screening platform. We then constructed expression plasmids for our *B. thetaiotaomicron* KDSR candidates and individually transformed them into our *Bt*SPT *E. coli* background. All candidate cultures were performed in minimal media, as yeast extract and other complex medium components contain sphingolipids. Interestingly, BT_0972 successfully converted 3-KDS to SA, where candidates BT_2380, BT_1433, and BT_3771 failed (Fig 2C, E). Indeed, supplementing the media with PA^alk^ led to the successful conversion of 3-ketosphinganine alkyne (3-KDS^alk^) to SA^alk^, further validating the activity of the gene product of *BT_0972* (Figs. 2, S5 and S6).

Previous attempts to characterize sphingolipid biosynthetic enzymes in bacteria using a candidate gene approach (9) or complementation libraries (21) have not led to the identification of the gene responsible for 3-KDSR gene activity in *Bacteroides*. Moreover, efforts to determine genes involved in *B. thetaiotaomicron* fitness estimate that *BT_0972* is essential (39). One may hypothesize that accumulation of 3-KDS may elicit toxicity as SPT-null strains of *B. thetaiotaomicron* are viable (10). Alternatively, BT_0972 may perform functions beyond sphingolipid biology, an observation further suggested by the lack of genetic clustering with SPT (*BT_0870*), a feature which may highlight additional functions, which do cluster with *BT_0972*. Additionally, previous attempts to remove 3-KDSR activity in *Saccharomyces* have failed (23). These observations suggest certain genetic approaches to characterize 3-KDSR activity in *B. thetaiotaomicron* could be challenging.

**Fig. 3:**
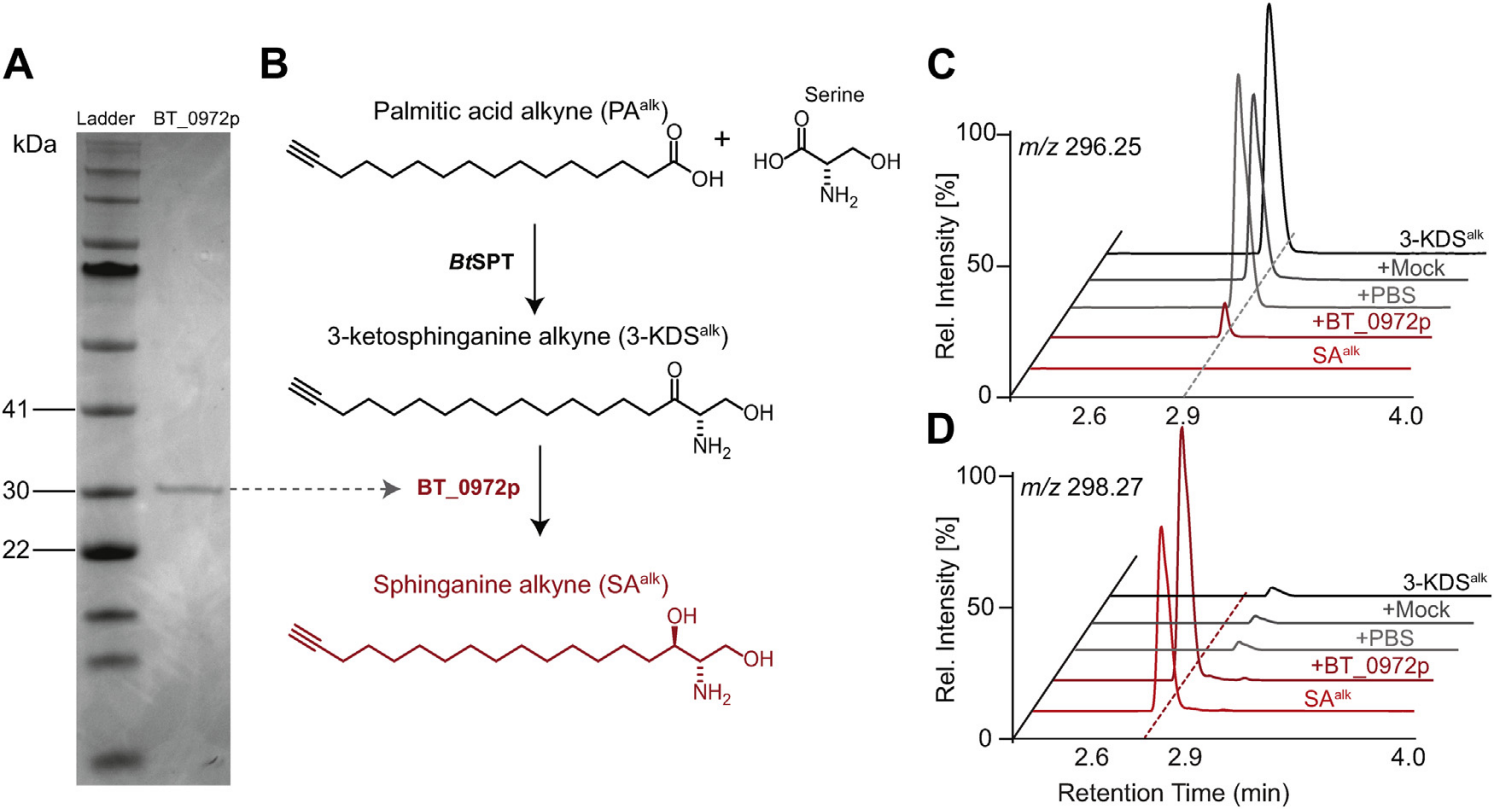
Enriched BT_0972 protein (BT_0972p) exhibits 3-KDSR activity. A: Enriched BT_0972p was analyzed by SDS-PAGE and stained with Coomassie Brilliant Blue. B: Scheme for sphingolipid biosynthesis. Mass spectrometry ion chromatograms of (C) 3-ketosphingaine alkyne (3-KDS^alk^) and (D) sphinganine alkyne (SA^alk^) demonstrating that the purified BT_0972p successfully produced SA^alk^. +PBS and +Mock are negative controls that represent vehicle and mock purification control respectively (n = 2 biological replicates per gene candidate and positive controls; representative traces are shown here.) 3-KDS^alk^, 3-ketosphinganine alkyne; 3-KDSR, 3-ketosphinganine reductase; SA^alk^, sphinganine alkyne.

Thus, we sought to investigate the *in vitro* 3-KDSR activity of BT_0972 via enzymatic methods. 3-KDS^alk^ was prepared using purified *Bt*SPT protein. Initial attempts to enrich recombinantly expressed hexahistidine-tagged BT_0972 in *E. coli* without detergent showed segregation of the protein to the insoluble fraction, suggesting either inclusion body accumulation or membrane association, or both. Indeed, previous attempts to isolate the human KDSR showed partitioning to membrane fractions (23). BT_0972 was therefore modestly solubilized via the addition of Triton X-100 to the lysis buffer and protein enrichment was performed via nickel-immobilized metal affinity chromatography. Purity of the enrichment was determined via SDS-PAGE, which resulted in the detection of a major band at ∼30 kDa (Fig. 3A, BT_0972p) matching the theoretical mass 30.99 kDa. In vitro–generated 3-KDS^alk^ (Fig. 3C, 3-KDS^alk^) was then incubated with enriched BT_0972p, which resulted in the predicted conversion to SA^alk^ (Fig. 3B–D) in an NADPH-dependent manner (supplemental Fig. S7). This provides further evidence that BT_0972 performs 3-KDSR activity in *B. thetaiotaomicron*.

### Phylogenetic analysis of 3-KDSR in sphingolipid-producing organisms

**Fig. 4:**
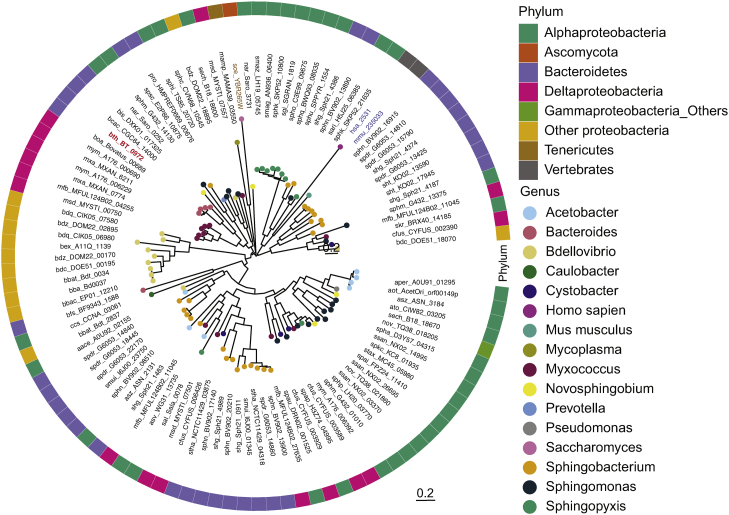
Bioinformatic and phylogenetic analysis identified KDSR homologs in known sphingolipid-producing bacteria. Phylogenetic tree based on BT_0972 and mammalian FVT-1 amino acid sequence comparisons are constructed using the neighbor-joining method from an alignment of currently known sphingolipid-producing bacteria. Tip nodes are colored by genus and the outer blocks are colored by phylum. Abbreviations can be found in supplemental Table S2. KDSR, ketosphinganine reductase.

We then attempted to identify BT_0972 homologs in other sphingolipid-producing bacterial systems (Fig. 4). As expected, the highest sequence similarities to BT_0972 were identified in other *Bacteroides* species, with *Prevotella*, another sphingolipid-producing human gut commensal, showing significant conservation. Additionally, we noted other sphingolipid-producing bacteria, *Bdellovibrio* and *Myxococcus*, showing considerable homology. The range in conservation of BT_0972 sequences between sphingolipid-producing bacteria suggests a diversity of biosynthetic strategies for de novo sphingolipid synthesis in bacteria.

Our data above reveal the identification and functional characterization of 3-KDSR in *B. thetaiotaomicron* (BT-KDSR). We endeavored to understand the phylogeny of BT-KDSR by BLAST analysis of the amino acid sequence of BT_0972 against a curated list of known sphingolipid-producing organisms (see methods for detail). The analysis generated affirmative alignment scores with an E-value cutoff ≤e-30. Further inspection of the aligned sequences established conservation of both the short-chain dehydrogenase/reductase family active site motif (Tyr-*X*-*X*-*X*-Lys) and the NADPH binding segment (Gly-*X*-*X*-*X*-Gly-*X*-Gly) (supplemental Fig. S8).

## Discussion

A fundamental understanding of the genes involved in bacterial sphingolipid synthesis is central to determining the importance of sphingolipids in mediating host-microbe interactions. These interactions influence phenotypes associated with host immunity and host metabolism. Although products of 3-KDSR activity have been measured in various species of bacteria (9, 10), the gene(s) responsible for this activity remained elusive. Identification of the cognate 3-KDSR is an essential step in revealing how bacterial sphingolipid synthesis modulates host-microbe interactions. Here, we describe 3-KDSR activity in *B. thetaiotaomicron* with generalizable application to related sphingolipid-producing taxa. We identified BT_0972 through a combination of comparative phylogenetics, *E. coli*-based heterologous expression strategies, and biochemistry to validate the transformation of 3-KDS to SA. Using this approach, we effectively engineered a non–sphingolipid-producing bacteria to produce a sphingolipid while simultaneously demonstrating the 3-KDSR activity of BT_0972. These findings are significant as they address attempts to identify the operative bacterial 3-KDSR. Prior to this investigation, it was proposed that *Bacteroides* performs a divergent de novo sphingolipid synthesis to that seen in eukaryotic systems. Our results highlight that the 3-KDSR activity is present in *B. thetaiotaomicron*, and certain *Bacteroides* species most likely synthesize SA using strategies like those observed in mammalian systems. This is different to what has been observed in other bacterial sphingolipid synthesis systems, therefore, adding to the evidence that bacterial sphingolipid synthesis varies across species. Understanding these differences may give insight into sphingolipid-dependent microbial fitness and how divergent bacterial sphingolipid synthesis strategies affect host-microbe interactions. Further work will define the roles associated with BT_0972 and how it contributes to the synthesis of specific bacterial lipids that modulate host-microbiome interactions.

## Data availability

All data are available in the manuscript.

## Supplemental data

This article contains supplemental data.

## Conflict of interest

The authors declare that they have no conflicts of interest with the contents of this article.

## Supplemental data

Lee-Le-Besler_JLR_sup table 2_Fig .4 taxa

Lee-Le-Besler_supplement_JLR_Revison
