# Supplementary material for: Identification and characterization of 3-ketosphinganine reductase activity encoded at the BT_0972 locus in Bacteroides thetaiotaomicron
Source: J Lipid Res. 2022 Jun 3;63(7):100236. doi: 10.1016/j.jlr.2022.100236 (PMC9278070; doi:10.1016/j.jlr.2022.100236)
Supplement: Lee-Le-Besler_JLR_sup table 2_Fig .4 taxa [file mmc1.docx]

---------------------

Score = 200.0

Length of alignment = 263

Sequence TSC10/5-251 (Sequence length = 320)

Sequence BT_2380/1-240 (Sequence length = 240)

TSC10/5-251 LEDQ--VVLITGGSQGLGKEFAKKYYNEAENTKIIIVSRSEARLLDTCNEIRIEAHLR

.| | . .|||.|||.| .| .. ......|| | . .. |

BT_2380/1-240 MEKQKGLAIITGASQGIGAVIAAGL--ATDGYRVVLIARSKQNL----EKVHDEI---

TSC10/5-251 RETTDEGQVQHKLAAPLDLEQRLFYYPCDLSCYESVECLFNALRDLDLLPTQTLCCAG

. .||. . |||. . | . | . . . .|.| . .

BT_2380/1-240 --MRSNKHVQEPIVLPLDITD------CTKADTE-IKDIHQKYGAVDILVNAAAMFMD

TSC10/5-251 GAVPKLFRGLSGHELNLGMDINYKTTLNVAHQIA-LAEQTKEHHLIIFSSATALYPFV

|. | . ... |.|| . . .. . |. .. .| .| | |

BT_2380/1-240 GS---LSE--PVDNFRKIMEINVIAQYGILKTVTEIMKVQKNGYIFNVASRAAKYGFA

TSC10/5-251 GYSQYAPAKAAIKSLVAILRQELT--NFRISCVYPG----NFESE---GFTVEQLTKP

. . |...| |. .| | .||. .|.. . || . | |.. .|

BT_2380/1-240 DGGIYGSTKFALLGLAESLYRELAPLGIRVTTLCPGWVNTDMAKKAGTPFKDEEMIQP

TSC10/5-251 E----ITKLIEGPSDAIPCKQACDIIAKSLA

. . . |. . |. . ||.

BT_2380/1-240 DDLLNTIRCLLNLSENVCIKDIVFEMKKSII

Percentage ID = 19.01

---------------------

Score = 430.0

Length of alignment = 286

Sequence TSC10/1-274 (Sequence length = 320)

Sequence BT_1433/3-270 (Sequence length = 270)

TSC10/1-274 MKFTLEDQVVLITGGSQGLGKEFAKKYYNEAENTKIIIVSRSEARLLDTCNEIRIEAH

| . |.||.||||. ||| .| | | .|.... ||| .|.

BT_1433/3-270 ELFNVKDKVVVITGGAGILGKGIAA--YLAKEGAKVVVLDRSE-----EAGKALVES-

TSC10/1-274 LRRETTDEGQVQHKLAAPLDLEQRLFYYPCDLSCYESVECLFNALRDLDLLPTQTLCC

.. | . . .| | . | .. |.|| . .

BT_1433/3-270 IKAEGN---EAMFLYTDVMDKEVLEGNKVEIMKAYGRIDVLLNAAGG-------NMAG

TSC10/1-274 AGGAVPKLFRGLSGHELNLGMDIN-YKTTLNVAHQIALAEQTKEHHLIIFSSATALYP

| | | | .| . .. .|.| . | | . . | .. | | .|| |

BT_1433/3-270 ATIAPDKTFFDLQIDAFKKVVDLNLFGTVLPTMVFAEIMVEQKKGSIVNFCSESALRP

TSC10/1-274 FVGYSQYAPAKAAIKSLVAILRQELT-----NFRISCVYPGNF---ESEG-FTV-E-Q

. |..||||| .. . ||. .|.. . || | .. . .| .

BT_1433/3-270 LTRVVGYGAAKAAIANFTKYMAGELALKFGNGLRVNAIAPGFFLTDQNRALLTNPDGS

TSC10/1-274 LTKPEITKLIEGPSDAIPCKQACDIIAKSLARGDDDVFTDFVGWMIMGMDLGLT

|| | | . | . . . | . . |. |. . | |

BT_1433/3-270 LTDRSKTILAHTPFNRFGEPEDLYGTIHYLISDASNFVTGTVAVIDGGFDAFSI

Percentage ID = 22.38

---------------------

Score = 340.0

Length of alignment = 276

Sequence TSC10/2-275 (Sequence length = 320)

Sequence BT_3771/1-248 (Sequence length = 248)

TSC10/2-275 KFTLEDQVVLITGGSQGLGKEFAKKYYNEAENTKIIIVSRSEARLLDTCNEIRIEAHL

|... ..||...|.|| .| |. || .. . | .. |. .

BT_3771/1-248 MGLLDGKTAIVTGAARGIGKAIALKF--AAEGAN---I----AF-----TDLVIDENA

TSC10/2-275 RRETTDEGQVQHKLAAPLDLEQRLFYYPCDLSCYESVECLFNALRDLDLLPTQTLCCA

. . . | . | . || . . . .|.| . |

BT_3771/1-248 EKTRVEL-EAMGVKAKGYA--SNAANFE-DTAKV--VEEIHKDFGRIDIL-VNN---A

TSC10/2-275 GGAVPKLFRGLSGHELNLGMDINYKTTLNVAHQIA-LAEQTKEHHLIIFSSATALYPF

| . | .| .. .. ...| |...| | . . . | .| .| ..

BT_3771/1-248 GITRDGLMMRMSEQQWDMVINVNLKSAFNFIHACTPVMMRQKAGSIINMASVVGVHGN

TSC10/2-275 VGYSQYAPAKAAIKSLVAILRQELTNFRISCVYPGNFESEGFTVEQLTKPEITKLIEG

| ..||..||.. .| . ||| . | .| . || . ..| . .

BT_3771/1-248 AGQANYAASKAGMIALAKSIAQELGSRGIR----ANAIAPGFILTDMTAALSDEVRAE

TSC10/2-275 PSDAIPCKQA-CDIIAKSLARGDDDVFTDFVGWMIMGMDLGLTA

. || ... ..| . .|. .. .| |.

BT_3771/1-248 WAKKIPLRRGGTPEDVANIATFLASDMSSYVSGQVIQVDGGMNM

Percentage ID = 17.75

---------------------

Score = 50.0

Length of alignment = 12

Sequence TSC10/1-12 (Sequence length = 320)

Sequence BT_0972/258-269 (Sequence length = 269)

TSC10/1-12 MKFTLEDQVVLI

|...| .

BT_0972/258-269 MQYALRIFYAIR

Percentage ID = 16.67

---------------------

Score = 2040.0

Length of alignment = 244

Sequence HUMAN_FVT1_3KDSR/28-255 (Sequence length = 332)

Sequence BT_2380/1-240 (Sequence length = 240)

HUMAN_FVT1_3KDSR/28-255 LALPGAHVVVTGGSSGIGKCIAIECYKQGAFITLVARNEDKLLQAKKE

. . ..||.| ||| || .| . |.||. ..| . |

BT_2380/1-240 MEKQKGLAIITGASQGIGAVIAAGLATDGYRVVLIARSKQNLEKVHDE

HUMAN_FVT1_3KDSR/28-255 IEMHSINDKQVVLCISVDVSQDYNQVENVIKQAQEKLGPVDMLVNCAG

| |.| .. | . ...|.. | . . ||. ..| |.||.||| |.

BT_2380/1-240 I-MRSNKHVQEPIVLPLDIT-DCTKADTEIKDIHQKYGAVDILVNAAA

HUMAN_FVT1_3KDSR/28-255 MAVSGKFEDLEVSTFERLMSINYLGSVYPSRAVITTMKERRVGRIVFV

| . | . . | | ..| || .. ..| || .. | | |

BT_2380/1-240 MFMDGSLSE-PVDNFRKIMEINVIAQYGILKTVTEIMKVQKNGYIFNV

HUMAN_FVT1_3KDSR/28-255 SSQAGQLGLFGFTAYSASKFAIRGLAEALQMEVKPYNVYITVAYP---

.|.|.. |. . |...|||. ||||.| |. | . .| |

BT_2380/1-240 ASRAAKYGFADGGIYGSTKFALLGLAESLYRELAPLGIRVTTLCPGWV

HUMAN_FVT1_3KDSR/28-255 -PDTD----TPGFAEENRTKP---LET-R-LISETTSVC-KPE--QVA

| || | .|. .| |.| | |.. . .|| | ..

BT_2380/1-240 NTDMAKKAGTP-FKDEEMIQPDDLLNTIRCLLNLSENVCIKDIVFEMK

HUMAN_FVT1_3KDSR/28-255 KQIV

| |.

BT_2380/1-240 KSII

Percentage ID = 30.33

---------------------

Score = 1070.0

Length of alignment = 281

Sequence **HUMAN**_FVT1_3KDSR/24-297 (Sequence length = 332)

Sequence BT_1433/1-270 (Sequence length = 270)

HUMAN_FVT1_3KDSR/24-297 SPKPLALPGAHVVVTGGSSGIGKCIAIECYKQGAFITLVARNEDKLLQ

. . . ||.|||.. .|| || |.|| . .. |.|.

BT_1433/1-270 MNELFNVKDKVVVITGGAGILGKGIAAYLAKEGAKVVVLDRSEE---A

HUMAN_FVT1_3KDSR/24-297 AKKEIEMHSINDKQVVLCISVDVSQDYNQVENVIKQAQEKLGPVDMLV

.| .| .... .. . || | . .| . | .|.|.

BT_1433/1-270 GKALVESIKAEGNEAMF-LYTDV-MDKEVLEGNKVEIMKAYGRIDVLL

HUMAN_FVT1_3KDSR/24-297 NCAG--MAVSG----K-FEDLEVSTFERLMSINYLGSVYPSRAVITTM

| || || . | | ||.. .| ... .| .|.| |. |

BT_1433/1-270 NAAGGNMAGATIAPDKTFFDLQIDAFKKVVDLNLFGTVLPTMVFAEIM

HUMAN_FVT1_3KDSR/24-297 KERRVGRIVFVSSQAGQLGLFGFTAYSASKFAIRGLAEALQMEVKPYN

|.. | || |... | .|.|.| || .. . |.

BT_1433/1-270 VEQKKGSIVNFCSESALRPLTRVVGYGAAKAAIANFTKYMAGELA---

HUMAN_FVT1_3KDSR/24-297 VYITVAYPPDTDTPGFAEENRTKPLETRLISETTSVCKPEQVAKQIVK

. . . .. .||| .. ..| | . | .. . .

BT_1433/1-270 LKFGNGLRVNAIAPGFFLTDQNRALLTNPDGSLTD--RSKTILAHTPF

HUMAN_FVT1_3KDSR/24-297 DAIQGNFNSSLGSDGYMLSALTCGMAPVTSITEGLQQVVTM

. |. . |. |..| . .. .. .| . ..

BT_1433/1-270 NR-FGEPEDLYGTIHYLISDASNFVTGTVAVIDGGFDAFSI

Percentage ID = 21.71

---------------------

Score = 1160.0

Length of alignment = 252

Sequence HUMAN_FVT1_3KDSR/27-275 (Sequence length = 332)

Sequence BT_3771/1-248 (Sequence length = 248)

HUMAN_FVT1_3KDSR/27-275 PLALPGAHVVVTGGSSGIGKCIAIECYKQGAFITLVARNEDKLLQAKK

| | .|||.. |||| ||. .|| |.. | . |

BT_3771/1-248 MGLLDGKTAIVTGAARGIGKAIALKFAAEGANIAFTDLVIDENAE-KT

HUMAN_FVT1_3KDSR/27-275 EIEMHSINDKQVVLCISVDVSQDYNQVENVIKQAQEKLGPVDMLVNCA

.|.... | . . . .... .|. . . .| .|.||| |

BT_3771/1-248 RVELEAMGVK--AKGYASN-AANFEDTAKVVEEIHKDFGRIDILVNNA

HUMAN_FVT1_3KDSR/27-275 GMAVSGKFEDLEVSTFERLMSINYLGSVYPSRAVITTMKERRVGRIVF

|.. | . . ....| .. .| | .. | |.

BT_3771/1-248 GITRDGLMMRMSEQQWDMVINVNLKSAFNFIHACTPVMMRQKAGSIIN

HUMAN_FVT1_3KDSR/27-275 VSSQAGQLGLFGFTAYSASKFAIRGLAEALQMEVKPYNVYITVAYPPD

..| | | | . |.||| .. .|| .. |. . . |

BT_3771/1-248 MASVVGVHGNAGQANYAASKAGMIALAKSIAQELGSRGIRANAIAPGF

HUMAN_FVT1_3KDSR/27-275 --TD-TPGFAEENRTKPLETRLISETTSVCKPEQVAKQIVKDAIQGNF

|| |.....| |. . . ..| . |

BT_3771/1-248 ILTDMTAALSDEVRAEWAKKIPLRRGGTPEDVANIATFLASDMSSYVS

HUMAN_FVT1_3KDSR/27-275 NSSLGSDGYMLS

. || |

BT_3771/1-248 GQVIQVDGGMNM

Percentage ID = 21.43

---------------------

Score = 1810.0

Length of alignment = 284

Sequence HUMAN_FVT1_3KDSR/30-298 (Sequence length = 332)

Sequence BT_0972/1-269 (Sequence length = 269)

HUMAN_FVT1_3KDSR/30-298 LPGAHVVVTGGSSGIGKCIAIECYKQGAFITLVARNEDKLLQAKKEIE

. ...||.|||.|| | || .. .| |

BT_0972/1-269 MQPQIILITGASSGFGKITAQMLSEQGHIVYGTSR---------KP--

HUMAN_FVT1_3KDSR/30-298 MHSINDKQVVLCISVDVSQDYNQVENVIKQAQEKLGPVDMLVNCAGMA

| | . | . |||.. . | . . | | .|.|.| |||.

BT_0972/1-269 --SENIGK-VRMLVVDVTNSIS-VRQAVEQIISEQGRMDVLINNAGMG

HUMAN_FVT1_3KDSR/30-298 VSGKFEDLEVSTFERLMSINYLGSVYPSRAVITTMKERRVGRIVFVSS

..| .| |. |..| | .||. |. | |.|. .||

BT_0972/1-269 IGGALELATEEEVSMQMNTNFFGVVNMCKAVLPYMRKARRGKIINISS

HUMAN_FVT1_3KDSR/30-298 QAGQLGLFGFTAYSASKFAIRGLAEALQMEVKPYNVYITVAYPPD---

.| .|. |||||||. | .||| .|| |... . . | |

BT_0972/1-269 IGGVMGIPYQGFYSASKFAVEGYSEALALEVHPFHIKVCLVQPGDFNT

HUMAN_FVT1_3KDSR/30-298 --TDT------PGFAEENRTKPL-ETRLIS-ETTSVCKPEQVAKQIVK

|| | |. | ..| | . | | ... | |

BT_0972/1-269 GFTDNRNISELTGQNEDYADSFLRSLKIIEKEERNGCHPRKLGAAICK

HUMAN_FVT1_3KDSR/30-298 DAIQGN--FNSSLGSDGYMLSALTCGMAPVTSITEGLQQVVTMG

. | | . .|. .| | . . | . .|. ..

BT_0972/1-269 IVARKNPPFRTKVGPLVQVLFAKSKSWLPDNMMQYALRIFYAIR

Percentage ID = 27.11

---------------------

Score = 1590.0

Length of alignment = 324

Sequence HUMAN_FVT1_3KDSR/26-327 (Sequence length = 332)

Sequence TSC10/1-320 (Sequence length = 320)

HUMAN_FVT1_3KDSR/26-327 KPLALPGAHVVVTGGSSGIGKCIAIECYKQG--AFITLVARNEDKLLQ

..| . |..|||| |.|| .| |... . | .|.|.| .||.

TSC10/1-320 MKFTLEDQVVLITGGSQGLGKEFAKKYYNEAENTKIIIVSRSEARLLD

HUMAN_FVT1_3KDSR/26-327 AKKEI--EMH----SINDKQVV--LCISVDVSQ---------D-YNQV

. .|| | | . .. || | ..|. | |. |

TSC10/1-320 TCNEIRIEAHLRRETTDEGQVQHKLAAPLDLEQRLFYYPCDLSCYESV

HUMAN_FVT1_3KDSR/26-327 ENVIKQAQE-KLGPVDMLVNCAGMAVSGKFEDLEVSTFERLMSINYLG

| ... .. | | . | ||| ||. | .| .. | |||

TSC10/1-320 ECLFNALRDLDLLPTQTLC-CAGGAVPKLFRGLSGHELNLGMDINYKT

HUMAN_FVT1_3KDSR/26-327 SVYPSRAVITTMKERRVGRIVFVSSQAGQLGLFGFTAYSASKFAIRGL

.. .. . . . . |.| || .. . |.. |...| ||..|

TSC10/1-320 TLNVAHQIALAEQTKEHHLIIF-SSATALYPFVGYSQYAPAKAAIKSL

HUMAN_FVT1_3KDSR/26-327 AEALQMEVKPYNVYITVAYPPDTDTPGFAEENRTKPLETRLISETTSV

|. |. | |. || . .. ||. |. ||| |.|| .

TSC10/1-320 VAILRQELT--NFRISCVYPGNFESEGFTVEQLTKPEITKLIEGPSDA

HUMAN_FVT1_3KDSR/26-327 CKPEQVAKQIVKDAIQGNFNSSLGSDGYMLSALTCGMAPVTSITEGLQ

| | | .|. . . | |. .. |... | ||

TSC10/1-320 IPCKQACDIIAKSLARGDDDVFTDFVGWMIMGMDLGLTAKKSRFVPLQ

HUMAN_FVT1_3KDSR/26-327 QVVTMGLFRTIALFYLGSFDSIVRRCMMQRE-KSEN

. . . ||. . .|. . . | |

TSC10/1-320 WIFGVLSNILVVPFYMVGCSWYIRKWFRENDGKKAN

Percentage ID = 25.62

**Supplemental Fig. 1 –** Pairwise sequence alignment between mammalian FVT-1, yeast TSC10, and 3-KDSR candidates in *Bacteroides thetaiotaomicron*.


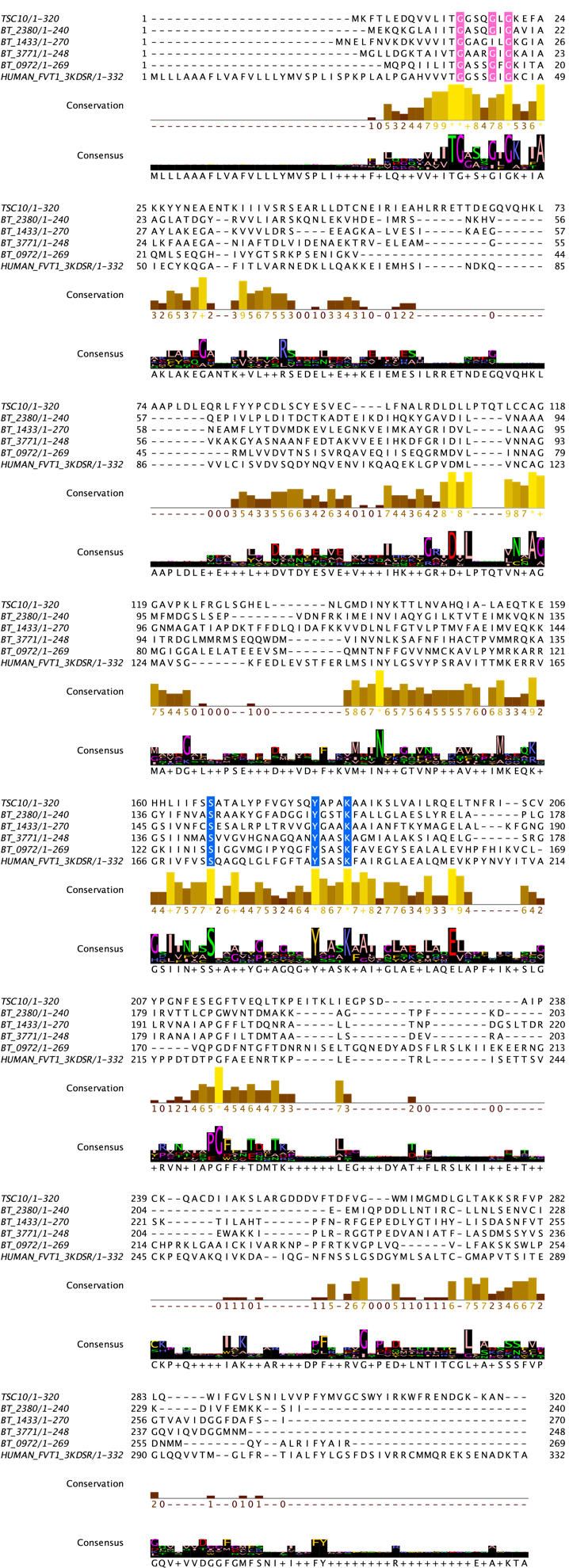


**Supplemental Fig. 2 –** Amino acid sequence alignment of the top four *B. thetaiotaomicron* 3-KDSR candidates comparing with TSC10 and FVT-1. The putative active site motif, Tyr-*X*-*X*-*X*-Lys (blue)

conserved in the short-chain dehydrogenase/reductase (SDR) family and the coenzyme NAD(H) or NADP(H) binding segment Gly-*X*-*X*-*X*-Gly-*X*-Gly (pink) are indicated.


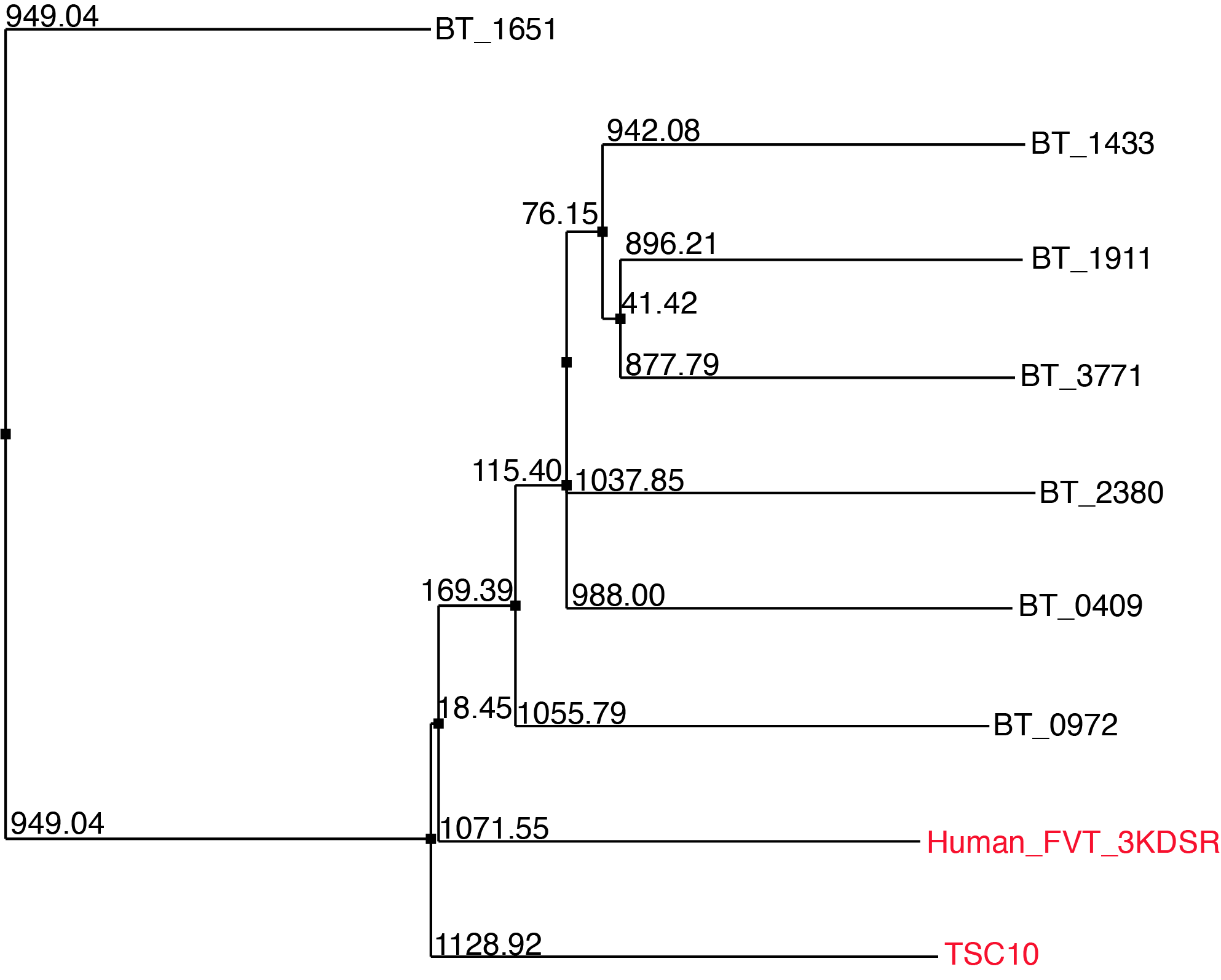


**Supplemental Fig. 3** – Phylogenetic tree of *B. thetaiotaomicron* 3-KDSR candidates and a mammalian FVT-1 and yeast TSC10 inferred from phylogenetic analysis of concatenated 3-KDSR gene orthologs. Numbers represent distance values for each branch based on the BLOSUM62 scoring matrix generated by Jalview.


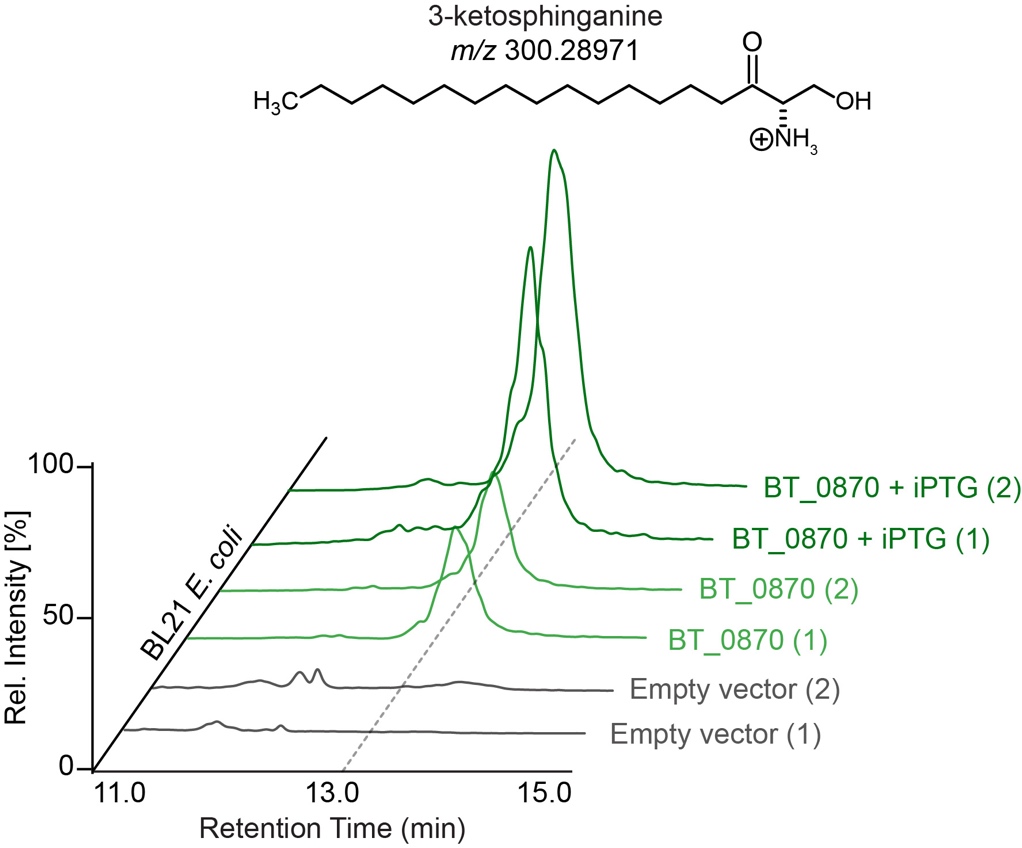


**Supplemental Fig. 4 –** Extracted high-resolution ion chromatograms of 3-ketosphinganine (3-KDS) produced in *E. coli* expressing the *B. thetaiotaomicron* SPT (BT_0870).


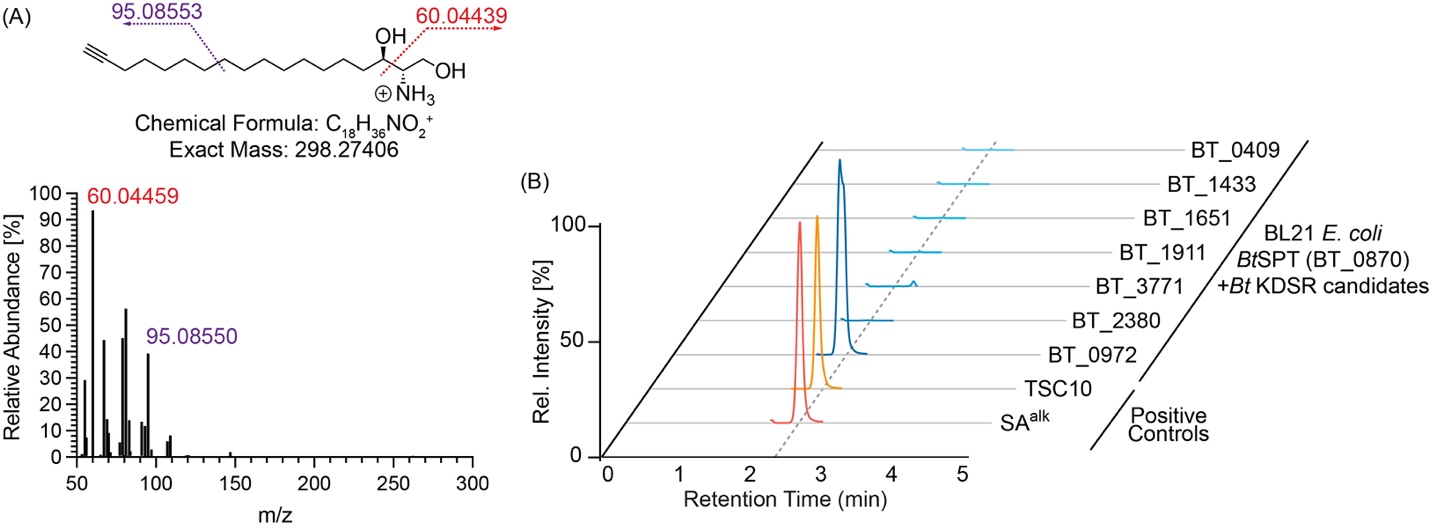


**Supplemental Fig. 5 -** (A) MS2 fragmentation patterns of sphinganine alkyne (SA^Alk^). (B) Ion chromatograms of the *B. thetaiotaomicron* KDSR candidates. *Saccharomyces cerevisiae* TSC10 and SA^Alk^ were used as positive control.


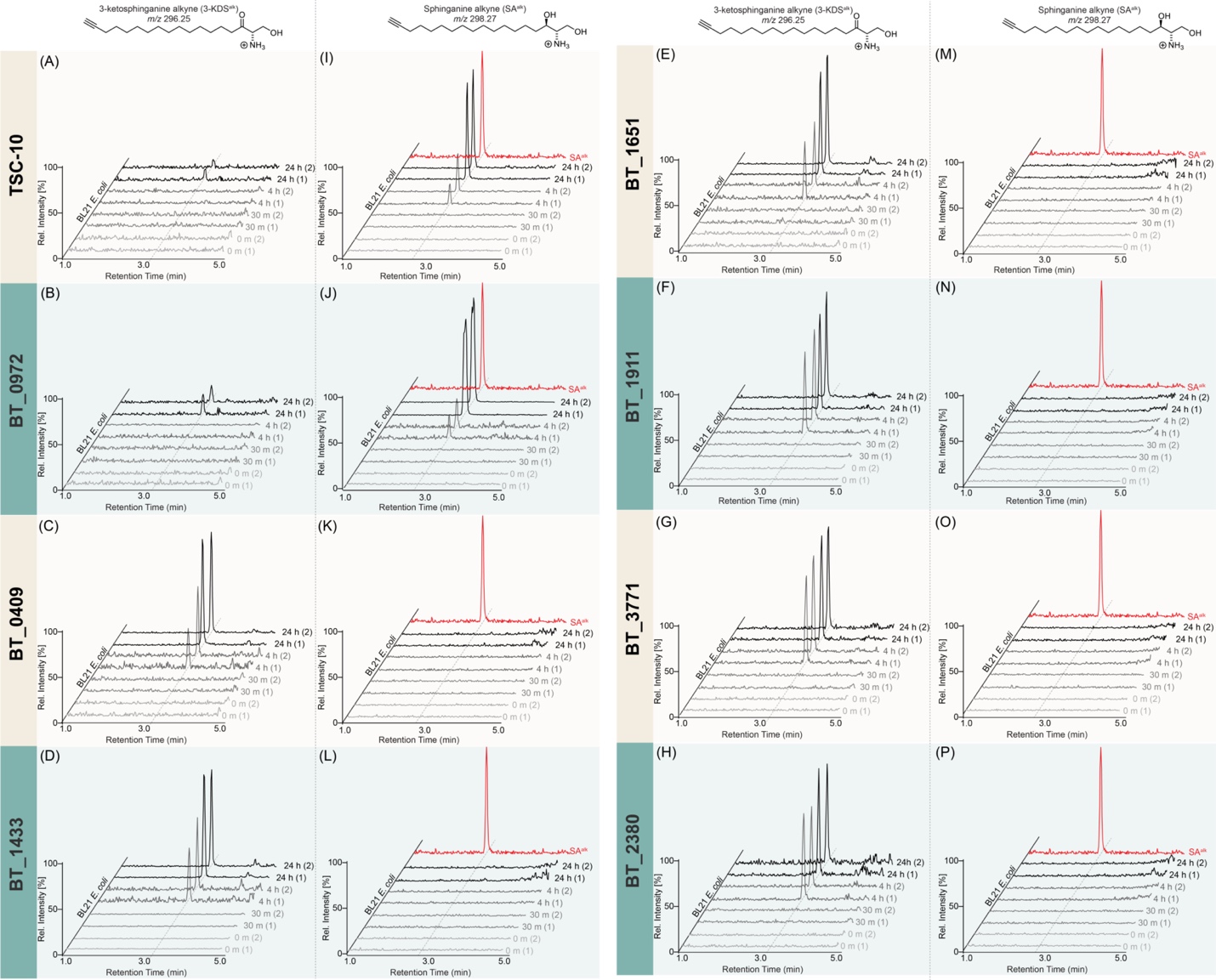


**Supplemental Fig. 6** - Ion chromatograms of (A-H) 3-ketosphinganine alkyne (3-KDS^alk^) and (I-P) sphinganine alkyne (SA^alk^) detected in *B. thetaiotaomicron* KDSR candidates that were heterologous expressed in BL21 *E. coli* and incubated with palmitic acid alkyne and *L*-serine over time. The incubation period (0 min, 30 min, 4 h and 24 h) is labeled on the right side of each chromatogram. (n = 2)


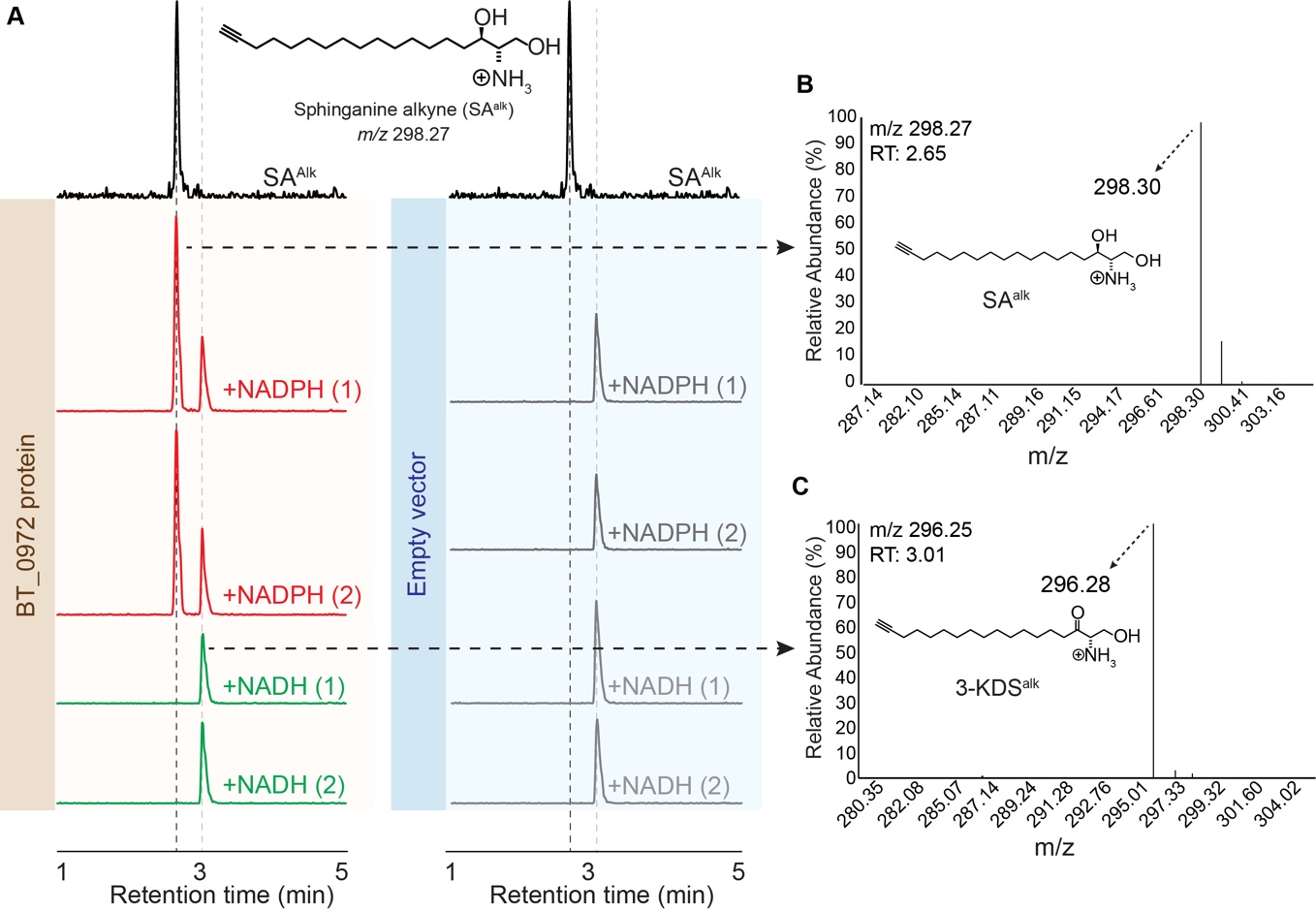


**Supplemental Fig. 7 - Enriched BT_0972 protein (BT_0972p) exhibits 3-KDSR activity in a NADPH-dependent manner.** (A) Extracted ion chromatograms (EICs) of sphinganine alkyne (SA^alk^) produced by BT_0972p in the presence or absence of NADPH or NADH as indicated and incubated at 37**°**C for 24h. Empty vector was subjected to the same incubation procedure to verify the results. EICs are shown on the same scale. (B and C) Selected range of spectrum corresponding to the arrow-pointed peak reveals SA^alk^ and 3-ketosphinganine alkyne (3-KDS^alk^) respectively.

**Supplemental Fig. 8 –** Multiple sequence alignment of homologs of BT_0972 in the known sphingolipid-producing bacteria and the representative eukaryotic sphingolipid producers (human, mouse and yeast). The alignments were derived using CLUSTALW built in the KEGG webpage and JalView. The putative active site motif, Tyr-*X*-*X*-*X*-Lys conserved in the SDR family (blue) and coenzyme NAD(H) or NADP(H) binding segment Gly-*X*-*X*-*X*-Gly-*X*-Gly (pink) are indicated.

| Gene | Primer name | Sequence |
| --- | --- | --- |
| BtSPT (BT_0870) | pET21_BT_0870_fwd | aattttgtttaactttaagaaggagatatacatATGGGATTATTACAAGAGAAGTTAGCT |
| BtSPT (BT_0870) | pET21_BT_0870_rev | ggatctcagtggtggtggtggtggtgctcgagCAAAAGGTCTAAAGCTTTGAAAGCTTTC |
| Sc_TSC10 | pET28_Sc_TSC10_fwd | gcatccatgggaATGAAGTTTACGTTAGAAGACCAAGTTG |
| Sc_TSC10 | pET28_Sc_TSC10_rev | cgtactcgagGTTGGCCTTCTTGCCGTC |
| BT_0972 | pET28_BT_0972 fwd | ctgccccatgggaATGCAACCACAAATCATACTCATTACC |
| BT_0972 | pET28_BT_0972 rev | cgtactcgagTCTGATAGCATAAAATATTCGGAGAGC |
| BT_1433 | pET28_BT_1433 fwd | ctgccccatgggaATGAACGAATTATTTAACGTAAAGGAC |
| BT_1433 | pET28_BT_1433 rev | cgtactcgagAATGGAGAATGCGTCGAAAC |
| BT_2380 | pET28_BT_2380_fwd | ttaactttaagaaggagatataccatgggaATGGAAAAGCAAAAAGGATTAGCTATTATT |
| BT_2380 | pET28_BT_2380_rev | atctcagtggtggtggtggtggtgctcgagAATAATGCTCTTTTTCATTTCAAAAACAAT |
| BT_3771 | pET28_BT_3771_fwd | ttaactttaagaaggagatataccatgggaATGGGATTATTAGACGGAAAAACAGCCATT |
| BT_3771 | pET28_ BT_3771_rev | atctcagtggtggtggtggtggtgctcgagCATATTCATACCACCATCTACCTGAATCAC |
| BT_0409 | pET28_BT_0409_fwd | ctgcccatgggaATGAAAGCGAAGATTGTTTTTATAACCG |
| BT_0409 | pET28_BT_0409_rev | gtggctcgagTTCTGCCTTCTTCTTATAAGAAATAGTGCC |
| BT_1651 | pET28_BT_1651_fwd | gcatccatgggaATGAGAAATATAGCAGTAATTCTAGCCG |
| BT_1651 | pET28_BT_1651_rev | cgtactcgagTCTTTTTACATCAACAACCTCACCTGTCATT |
| BT_1911 | pET28_BT_1991_fwd | ttggccatgggaATGAGAAAGATCGATTTAATCGTGATCC |
| BT_1911 | pET28_BT_1991_rev | ggttctcgagCAGGCTTTCGTTTACCTCAAAGC |

**Supplemental Table 1 –** Primers used in this study
